# Association between dietary total antioxidant capacity and hypertension in Iranian Kurdish women

**DOI:** 10.1186/s12905-022-01837-4

**Published:** 2022-06-25

**Authors:** Hawal Lateef Fateh, Narmin Mirzaei, Mohammed Ibrahim Mohialdeen Gubari, Mitra Darbandi, Farid Najafi, Yahya Pasdar

**Affiliations:** 1 Nursing department, Sulaimani Polytechnic University, Sulaimani, 46001 Kurdistan region Iraq; 2 Student Research Committee, Kermanshah University of Medical Sciences, Kermanshah, Iran; 3 Department of Family and Community Medicine, College of Medicine, University of Sulaimani, Sulaimani, 46001 Kurdistan region Iraq; 4 Research Center for Environmental Determinants of Health (RCEDH), Health Institute, Kermanshah University of Medical Sciences, Kermanshah, Iran; 5 Cardiovascular Research Center, Kermanshah University of Medical Sciences, Kermanshah, Iran

**Keywords:** Dietary, Total antioxidant capacity, Hypertension, Persian

## Abstract

**Background:**

Antioxidants intake from diet has been identified as one of the effective factors in the development of hypertension (HTN). The present study aimed to investigate the association between total antioxidant capacity (TAC) and HTN in women.

**Methods:**

This cross-sectional study was performed using the baseline phase data of the ravansar non-communicable disease cohort study. The TAC was calculated using food items of the food frequency questionnaire. TAC scores were classified into four groups (quartile). The first and fourth quartiles had the lowest and highest TAC scores, respectively. Logistic regression analysis was utilized to estimate the odds ratio.

**Results:**

A total of 5067 women were included in the study. Women with the highest socioeconomic status (SES) had a significantly higher TAC intake compared to those with the lowest SES (*P* < 0.001). The participants in the third and fourth quartiles of the TAC had significantly lower odds of HTN, respectively by 21% (OR = 0.79; 95% CI: 0.64, 0.972) and 26% (OR = 0.74; 95% CI: 0.60, 0.91), compared to the first quartile. After adjusting for confounding variables was found to significantly reduce the odds of developing HTN in the fourth quartile of TAC by 22% compared to the first quartile (OR = 0.78; 95% CI: 0.62, 0.97).

**Conclusion:**

A high dietary TAC was associated to a decreased odd of HTN in women. We could suggest a diet rich in natural antioxidants as it may help prevent development of HTN.

## Introduction

Hypertension (HTN) is a common non-communicable disease (NCDs) with an increasing prevalence, especially in low- and middle-income countries [1, 2]. A systematic review and meta- analysis study reported that the prevalence of HTN is 22% (men: 23.6% and women: 23.5%) in Iran [3]. Studies on middle-aged people have shown that the prevalence of HTN is higher in post-menopausal women compared to that in men [4, 5].

HTN is a multifactorial disease that includes both modifiable and non-modifiable risk factors [1, 2]. One of the factors that causes NCDs, especially HTN, is oxidative stress. Oxidative stress is a key factor in the pathogenesis of chronic diseases through free radicals, leading to biological damage [6]. Oxidative stress, by creating an imbalance between peroxidation and antioxidants, leads to potential changes in endothelial cells and can act as an auxiliary mechanism in causing high blood pressure [7]. High dietary total antioxidant capacity (TAC) can reduce oxidative stress by inhibiting free radicals [8, 9]. Therefore, dietary pattern is one of the risk factors for HTN. Previous research has demonstrated an association between food groups and TAC with HTN [10–12]. Moreover, a study on French women has demonstrated that a high antioxidant capacity was associated with a reduced risk of incident HTN [13]. Additionally, previous papers have shown an inverse association between dietary antioxidants and the risk of CVDs and metabolic syndrome (MetS) [7, 14]. On the other hand, scientific evidence has shown that an inflammatory diet is correlated with an increased risk of developing type 2 diabetes, hypertension, cardiovascular diseases (CVDs), and non-alcoholic fatty liver (NAFLD) [15–17]. According to a review study, antioxidants have anti-inflammatory effects although different types of antioxidants may have different effects since they are structurally and chemically different [7].

According to the mechanism of antioxidants in causing oxidative stress, particularly in women, it is hypothesized that antioxidants are associated with HTN in women. Accordingly, the objective of the current study was to assess the association between dietary TAC and HTN in Iranian Kurdish women.

## Methods

### Study design and population

This cross-sectional study was performed using the baseline phase data of the Ravansar Non-Communicable disease (RaNCD) prospective cohort study. The RaNCD study is one of the prospective epidemiological research studies in Iran (PERSIAN). Ravansar is a district with urban and rural areas, which is located in the west of Iran and in Kermanshah province with a population of about 50,000. The initial phase of the RaNCD study began in 2014 with the enrolment of 10,047 adults aged 35–65 and has continued to date. The complete methodology of the RaNCD study has already been published [18].

All the women participating in the baseline phase of the RaNCD research were included in the study (n = 5283). The pregnant women, cancer patients, and those with energy intake less than 800 kcal/day or more than 4200 kcal/day were excluded. Finally, 5067 women were examined in this study.

### Data collection

Questionnaire information was collected in the form of face-to-face interviews by trained experts. The SES index was measured, using 18 items, including education level, residential place, and wealth indicators with principal component analysis (PCA) method, subsequent to which SES was categorized into five groups, from the lowest to the highest.

Lifestyle-associated factors included smoking status and physical activity. The level of physical activity was assessed using the PERSIAN cohort questionnaire [19], which consists of 22 questions, classified as low (24–36.5 MET/hour per day), moderate (36.6–44.4 MET/hour per day), and high (≥ 44.5 MET/hour per day). In addition, current-smokers were the participants who reported they had smoked at least 100 cigarettes.

Height was measured using a BSM 370 (Biospace Co, Seoul, Korea). Body weight was measured using a Bio Impedance Analyser BIA (InBody 770 Biospace, Korea), waist-to-hip ratio (WHR) was calculated by dividing the waist circumference by hip circumference. Blood pressure was estimated with a manometer (Reister) cuff and stethoscope (Reister) in the seated position after they had rested for at least 10 min.

### Assessment of dietary total antioxidant capacity

Dietary information was collected using a national validated semi-quantitative food frequency questionnaire (FFQ) with 118 items, which was done face-to-face. The participants responded the questions about the frequency and the amount of food consumed (portion size), including bread and cereals, dairy, red and white meat, legumes, fruits and vegetables, sweets and desserts, tea and coffee, as well as nuts and oils and fats [20]. A booklet with standard values was used to help estimate the portion size.

The TAC was calculated by multiplying the amount received from each food item (in grams) based on the amount of antioxidants per gram of food (mol/100 grµ). The method used to estimate dietary TAC was ORCA and its unit was reported in mmol. The amount of antioxidant capacity for each food item was calculated separately and then TAC was obtained from the sum of the amount of antioxidant capacity received from all the food items. The TAC content of each food item was calculated in the second version of the TAC content of food parameters published by the USDA and study of Wu et al. [21, 22]. Finally, TAC scores were classified into four groups (quartile). The first and fourth quartiles had the lowest and highest TAC scores, respectively.

### Definitions

HTN was defined as having a systolic blood pressure (SBP) ≥ 140 mmHg and/or diastolic blood pressure (DBP) ≥ 90 mmHg, or taking antihypertensive medication. Dyslipidaemia was defined as total cholesterol (mg/dl) ≥ 240 and/or LDL cholesterol (mg/dL) ≥ 160 and/or HDL cholesterol (mg/dL) < 40 and/or triglycerides (mg/dL) ≥ 200 and/or having a history of medication for lipid disorders [23]. Menopause status was defined as having no menstrual for the last 12 months. Women having menstruated for the past 12 consecutive months were defined as pre/perimenopausal, and those with no menstruation for the past 12 months were defined as menopausal.

### Statistical analyses

Regarding the descriptive section of this study, quantitative variables by mean ± standard deviation indices are presented and qualitative variables are reported in frequency (percentage). We categorized the participants into quartiles according to TAC intake, and considered the lowest group as a reference. The normality of the data was assessed using the Kolmogorov–Smirnov test. In addition, mean ± standard deviation of the anthropometric and biochemical characteristics was compared by one-way ANOVA among the four studied groups, and frequency (percent) was compared by Chi square test. The mean ± standard deviation of food parameters was compared with t-test between hypertensive and non- hypertensive groups. Crude and adjusted logistic regression model was used for determining the association between the risk factors and HTN, and estimates were reported with 95% confidence interval. In the adjusted model, we controlled the potentially confounding variables, including age, energy intake, physical activity, menopausal status, Body mass index (BMI), SES, and smoking. For statistical analyses, a *P*-value of < 0.05 with 95% confidence intervals (CIs) was considered to be significant. All the analyses were carried out with STATA software version 14.2 (Stata Corp, College Station, Tex).

## Results

A total of 5067 women aged 35–65 years were analysed in this study, out of whom 69.09% (3501) were in pre/perimenopausal and 30.91% (1566) were in menopausal. Table 1 represents the basic characteristics of the participants based on TAC quartiles. The women included in the highest quartile of TAC were significantly younger than those in the lowest quartile (*P* < 0.001). Compared to the lower SES levels, dietary TAC intake was significantly higher at higher SES levels (*P* < 0.001). The prevalence of HTN was significantly lower in the women with higher TAC intake (*P* = 0.023).

**Table 1:** Baseline characteristics of the study population according to total antioxidant capacity quartiles (RaNCD cohort data, N = 5067)

Variable	Dietary total antioxidant capacity (TAC)				*P* value*
	Q1 n = 1569	Q2 n = 1295	Q3 n = 1.121	Q4 n = 1082	
	Mean ± SD/n (%)				
TAC score	0.10 ± 0.02	0.16 ± 0.02	0.24 ± 0.03	0.46 ± 0.19	–
Age	49.07 ± 8.68	47.38 ± 8.28	46.99 ± 8.35	46.52 ± 8.06	< 0.001
BMI (kg/m^2^)	27.69 ± 4.83	28.71 ± 4.94	28.88 ± 4.90	29.17 ± 4.69	< 0.001
WHR	0.94 ± 0.05	0.94 ± 0.06	0.95 ± 0.06	0.95 ± 0.06	< 0.001
*Marital status*					
Married	1234 (78.65)	1099 (84.86)	972 (86.71)	938 (86.69)	< 0.001
Single	139 (8.86)	67 (5.17)	66 (5.89)	50 (4.62)	
Widowed/Divorced	196 (12.49)	129 (9.96)	83 (7.40)	94 (8.69)	
*Socio-economic status*					
1(lowest)	781 (49.78)	430 (33.20)	285 (25.47)	192 (17.76)	< 0.001
2	543 (34.17)	471 (36.37)	362 (32.35)	312 (28.86)	
3(highest)	245 (15.62)	394 (30.42)	472 (42.18)	577 (53.38)	
*Physical activity (Met-h/day)*					
Light	412 (26.26)	357 (27.57)	271 (24.17)	283 (26.16)	< 0.001
Moderate	973 (62.01)	768 (59.31)	742 (66.19)	713 (65.90)	
High	184 (11.73)	170 (13.13)	108 (9.63)	86 (7.95)	
*Menopausal status*					
Pre/perimenopausal	985 (62.78)	912 (70.42)	808 (72.08)	796 (73.57)	< 0.001
Post-menopausal	584 (37.22)	383 (29.58)	313 (27.92)	286 (26.43)	
Current smoker	37 (2.38)	21 (1.64)	25 (2.26)	15 (1.40)	0.010
Hypertension	296 (18.87)	217 (16.76)	174 (15.52)	159 (14.70)	0.023

*BMI* Body mass index; *WHR* Waist hip ratio

*Analysis of variance (ANOVA) and Chi square, *P* < 0.05

Table 2 depicts the daily intake of macro- and micro-nutrients according to TAC quartiles. Daily intake of dairy, fruit, legumes, vegetables, sea food, egg, omega-3, omega-6, and vitamins of A, B1, B2, B6, B12, C, D, and E were significantly higher in the highest quartile of TAC after adjust for energy intake (*P* < 0.001).

**Table 2:** Nutrients intake according to the dietary antioxidant capacity of participants (RaNCD cohort data, N = 5067)

Food parameters	Dietary total antioxidant capacity (TAC) Mean ± SD				*P* value*
	Q1 n = 1575	Q2 n = 1.299	Q3 n = 1.123	Q4 n = 1085	
Energy intake (kcal/d)	1597.85 ± 584.24	1892.16 ± 601.00	2047.03 ± 667.45	2277.61 ± 773.14	< 0.001
Carbohydrate (%E)	68.60 ± 7.26	65.49 ± 7.24	63.32 ± 7.46	61.17 ± 8.01	< 0.001
Protein (%E)	15.38 ± 1.91	15.75 ± 2.13	15.81 ± 2.07	15.95 ± 2.17	< 0.001
Lipid (%E)	15.62 ± 6.54	18.56 ± 6.66	20.81 ± 7.08	23.00 ± 7.73	< 0.001
Whole grains (gr/d)	26.85 ± 1.41	32.24 ± 1.50	37.64 ± 1.62	47.26 ± 1.70	< 0.001
Refined grains (gr/d)	580.98 ± 4.17	535.80 ± 4.43	501.36 ± 4.79	443.36 ± 5.01	< 0.001
Legumes (gr/d)	25.76 ± 0.72	34.06 ± 0.77	42.39 ± 0.82	53.91 ± 0.86	< 0.001
Dairy (gr/d)	378.89 ± 9.23	423.73 ± 9.81	442.86 ± 10.58	472.42 ± 11.10	< 0.001
Fruit (gr/d)	96.96 ± 4.24	183.46 ± 4.50	265.99 ± 4.86	400.77 ± 5.10	< 0.001
Vegetables (gr/d)	371.40 ± 6.89	474.16 ± 7.32	562.45 ± 7.90	666.16 ± 8.26	< 0.001
Red meat (gr/d)	28.53 ± 0.79	34.03 ± 0.84	36.24 ± 0.90	40.70 ± 0.95	< 0.001
Poultry (gr/d)	3.47 ± 0.19	3.99 ± 0.21	4.21 ± 0.22	2.93 ± 0.23	< 0.001
Sea food (gr/d)	0.74 ± 0.10	1.23 ± 0.10	1.53 ± 0.10	1.82 ± 0.11	< 0.001
Egg (gr/d)	1.65 ± 0.18	2.81 ± 0.19	4.19 ± 0.21	6.63 ± 0.22	< 0.001
Salt (gr/d)	4.19 ± 0.10	4.25 ± 0.10	4.20 ± 0.08	4.23 ± 0.10	0.928
Nuts (gr/d)	27.30 ± 0.82	32.85 ± 0.87	33.15 ± 0.94	32.34 ± 0.98	< 0.001
Tea & coffee (gr/d)	1.43 ± 2.78	2.67 ± 4.45	3.76 ± 6.82	4.95 ± 9.30	< 0.001
Olive oil (gr/d)	0.15 ± 0.05	0.38 ± 0.05	0.72 ± 0.06	1.30 ± 0.06	< 0.001
Dried fruits (gr/d)	22.37 ± 0.62	25.50 ± 0.66	27.42 ± 0.71	30.76 ± 0.74	< 0.001
Juices fruits (gr/d)	0.24 ± 0.01	0.47 ± 0.05	0.67 ± 0.06	0.89 ± 0.06	< 0.001
Omega-3 (mcg)	0.08 ± 0.01	0.11 ± 0.02	0.12 ± 0.02	0.14 ± 0.02	< 0.001
Omega- 6 (mcg)	0.23 ± 0.01	0.30 ± 0.01	0.34 ± 0.1	0.35 ± 0.02	< 0.001
Vitamin A (mcg)	425.14 ± 12.03	580.11 ± 12.78	710.45 ± 13.80	879.40 ± 14.43	< 0.001
Vitamin B1 (mg)	0.41 ± 0.01	0.58 ± 0.01	0.71 ± 0.01	0.89 ± 0.01	< 0.001
Vitamin B2 (mg)	0.54 ± 0.01	0.76 ± 0.01	0.93 ± 0.02	1.13 ± 0.01	< 0.001
Vitamin B6 (mg)	0.74 ± 0.01	1.01 ± 0.01	1.20 ± 0.01	1.44 ± 0.01	< 0.001
Vitamin B12 (mcg)	2.10 ± 0.10	2.63 ± 0.10	3.16 ± 0.10	3.68 ± 0.10	< 0.001
Vitamin C (mg)	44.33 ± 0.96	56.87 ± 1.03	67.72 ± 1.11	87.98 ± 1.15	< 0.001
Vitamin D (mcg)	0.69 ± 0.01	0.85 ± 0.01	1.06 ± 0.01	1.18 ± 0.01	< 0.001
Vitamin E (mg)	1.53 ± 0.03	2.13 ± 0.03	2.51 ± 0.04	2.99 ± 0.04	< 0.001

The mean ± SD of food groups and micronutrients is adjusted for daily energy intake

*Analysis of variance (ANOVA), *P* < 0.05

The mean daily intake of refined grains, legumes, white meat, omega-3, omega-6, and vitamin D were significantly higher among the non-hypertensive women compared to the hypertensive ones after adjust for energy intake (Table 3).

**Table 3:** Comparison of dietary pattern in participants with and without hypertension (RaNCD cohort data, N = 5067)

Food parameters	Non-hypertension	Hypertension	*P* value*
	Mean ± SD		
Whole grains (gr/d)	33.61 ± 0.84	39.45 ± 1.87	0.004
Refined grains (gr/d)	526.40 ± 2.55	504.25 ± 5.73	< 0.001
Legumes (gr/d)	38.60 ± 0.44	35.40 ± 1.10	0.011
Dairy (gr/d)	450.33 ± 5.45	419.21 ± 12.21	0.021
Fruit (gr/d)	220.71 ± 2.97	234.64 ± 6.65	0.031
White meat (gr/d)	19.41 ± 0.11	15.52 ± 0.25	< 0.001
Red meat (gr/d)	34.16 ± 0.47	34.61 ± 1.05	0.702
Sea food (gr/d)	1.27 ± 0.05	1.23 ± 0.11	0.742
Egg (gr/d)	3.59 ± 0.11	3.48 ± 0.24	0.664
Soda drink (gr/d)	1.79 ± 0.06	1.42 ± 0.13	0.009
Leafy vegetables (gr/d)	360.80 ± 3.50	370.58 ± 7.80	0.127
Carotene rich vegetables (gr/d)	88.42 ± 1.36	91.36 ± 3.04	0.340
Starchy vegetables (gr/d)	42.46 ± 0.71	43.90 ± 1.60	0.571
Nuts (gr/d)	31.77 ± 0.48	29.02 ± 1.10	0.138
Tea & coffee (gr/d)	3.20 ± 6.31	2.11 ± 5.19	< 0.001
Olive oil (gr/d)	0.56 ± 0.03	0.67 ± 0.07	0.157
Dried fruits (gr/d)	26.35 ± 0.36	25.72 ± 0.82	0.562
Juices fruits (gr/d)	0.55 ± 0.03	0.42 ± 0.07	0.067
Omega-3 (mcg)	0.11 ± 0.08	0.10 ± 0.07	0.008
Omega- 6 (mcg)	0.30 ± 0.04	0.28 ± 0.01	0.043
Vitamin A (mcg)	622.10 ± 7.48	637 ± 16.76	0.407
Vitamin B1 (mg)	0.61 ± 0.01	0.63 ± 0.01	0.112
Vitamin B2 (mg)	0.81 ± 0.01	0.80 ± 0.01	0.815
Vitamin B6 (mg)	1.06 ± 0.01	1.04 ± 0.01	0.201
Vitamin B12 (mcg)	2.79 ± 0.04	2.87 ± 0.10	0.467
Vitamin C (mg)	60.46 ± 0.06	63.10 ± 1.35	0.121
Vitamin D (mcg)	0.92 ± 0.01	0.83 ± 0.02	< 0.001
Vitamin E (mg)	2.27 ± 0.02	2.34 ± 0.04	0.220

The mean ± SD of food groups and micronutrients is adjusted for daily energy intake

*Using t-test, *P* < 0.05

Table 4 illustrates the association between the dietary total antioxidant capacity (TAC) and the HTN by logistic regression analysis. The individuals in the third and fourth quartiles of the diet TAC had a significantly lower odd of HTN, respectively by 21% and 26%, compared to the first quartile (Model 1). After adjustment of menopausal status, this association remained significant in the fourth quartile (OR = 0.81; 95% CI: 0.68, 0.98). After adjustment of potential confounding, including energy intake, SES, BMI, physical activity, and menopausal status, were found to significantly reduce the odds of developing HTN in the fourth quartile of TAC by 22% compared to the first quartile (Model 3).

**Table 4:** Association between the dietary total antioxidant capacity (TAC) and the hypertension by logistic regression analysis

Models regression		Quartiles of total antioxidant capacity (TAC)				P value trend
		Quartile 1	Quartile 2	Quartile 3	Quartile 4	
Model I	OR (95% CI)	1 (Ref.)	0.86 (0.71, 1.05)	079 (0.64, 0.972)	0.74 (0.60, 0.91)	0.003
	*P* value	–	0.143	0.025	0.005	
Model II	OR (95% CI)	1 (Ref.)	0.95 (0.78, 0.16)	0.88 (0.71, 1.09)	0.84 (0.68, 1.05)	0.105
	*P* value	–	0.646	0.270	0.136	
Model III	OR (95% CI)	1 (Ref.)	0.93 (0.75, 1.14)	0.84 (0.68, 1.05)	0.78 (0.62, 0.97)	0.020
	*P* value	–	0.504	0.123	0.028	

Model I: Crude; Model II: Adjusted for menopausal status; Model III: Adjusted for age, menopausal status, energy intake, SES, BMI, physical activity and smoking

## Discussion

In the present study, we found a significant association between higher dietary TAC intake and reduced odds of HTN. After adjustment of age, energy intake, SES, BMI, physical activity, and menopausal status, this association remained significant, and the odds of developing HTN in the top quartile of TAC by 22% was lower compared to the lowest quartile. Consistent with the findings of this study, previous research has demonstrated the association between food groups, including fruits and vegetables, and TAC with HTN [10–13].

According to evidence, oxidative stress is caused by the overproduction of oxygen-free radicals or a decrease in the concentration of antioxidants in the body [7]. On the other hand, HTN is indirectly the result of an imbalance of antioxidants to inhibit free radicals [7, 24]. Meta-analysis studies have shown that increased intake of fruits, vegetables, and vitamins is correlated with a reduced risk of developing HTN [10, 12]. Our findings also revealed that the intake of legumes, nuts, tea and coffee, fruit juice, and omega 3, omega 3, and vitamins of B1, B2, B6, and D were significantly lower in the women with HTN. Only one cohort study on French women reported a significant association between dietary TAC and HTN [13]. Ahmad et al. [25] suggested that the antioxidant therapy to reduce oxidative stress is a promising strategy for prevention and treatment of cardiovascular events, involving HTN. However, in a clinical trial study, no association was found between antioxidant supplementation and a reduction in HTN [26]. This may be due to the fact that the intervention was supplements. Therefore, it is better to devise interventions on diet change rather than supplements. On the other hand, it is important to note that numerous antihypertensive drugs are currently used in clinical practice, such as calcium channel blockers, β-blockers, angiotensin-converting enzyme inhibitors, and angiotensin receptor blockers; in addition to reducing blood pressure, adrenergic can decline the antioxidant effects and activity of several vascular matrix metalloproteinase [27]. Similarly, in hypertensive rats, antihypertensive drugs, such as losartan, nifedipine, metoprolol, nebivolol, and nimodipine, have been shown to reduce vascular oxidative stress and matrix metalloproteinase expression; thus, they have an antioxidant effect [28, 29]. Overall, these findings supported the theory that HTN can be treated with dietary/natural antioxidants. However, clinical trial studies are extremely necessary to determine the responsibility of oxidative stress on HTN and the possible therapy of high blood pressure with antioxidants.

We found that women with the most intake of antioxidant had a significantly lower LDL-C and TC. Dyslipidaemia is one of the most important risk factors for HTN [30]. A study by Kashyap et al. showed that blood lipid levels were higher in subjects with high blood pressure compared to those with normal blood pressure [31]. The plasma malondialdehyde (MDA) increases in subjects with hypertension, MDA is the final product of non-enzymatic degradation of polyunsaturated fatty acids (PUFA). In fact, higher levels of MDA in hypertensive individuals increase lipid peroxidation and the production of peroxides in the cell membrane, which leads to the production of free radicals. Free radicals also cause dyslipidaemia and raise uric acid and plasma homocysteine, which is why subjects with HTN have dyslipidaemia [31]. Therefore, a diet rich in antioxidants may have a double role in the prevention of HTN.

The strengths of this study include the followings: large sample size, use of RaNCD prospective study data, and use of a valid questionnaire with 118 food items to calculate dietary TAC. This research is the first study in Iran on a large population of women of Kurdish ethnicity. One of the limitations of this study is its cross-sectional nature that was not able to present causal associations. There were measurement errors encountered in collecting the nutritional information. Furthermore, in the food frequency questionnaire used in this study, local foods were also included; therefore, to prove the generalizability of the results, it is necessary to conduct further research in different regions and with different dietary patterns.

## Conclusion

The findings of this study demonstrated a significant association between a high dietary TAC and reduced odds of HTN among Iranian Kurdish women. We could suggest a diet rich in natural antioxidants as it may help prevent the development of HTN from younger ages. Nonetheless, these results need to be interpreted with caution. To definitively accept the results of this study, further investigation is needed on different demographic and ethnic subgroups.

## Data Availability

The data analysed in the study are available from the corresponding author on reasonable request.

## Abbreviations

HTN: Hypertension
CVDs: Cardiovascular diseases
FFQ: Food frequency questionnaire
NCDs: Common non-communicable disease
SES: Socioeconomic status
TAC: Total antioxidant capacity
RaNCD: Ravansar non-communicable disease
NAFLD: Non-alcoholic fatty liver
FBS: Fasting blood sugar
HDL-C: High-density lipoprotein cholesterol
LDL-C: Low-density lipoprotein cholesterol
TC: Total cholesterol
TG: Triglyceride
PERSIAN: Prospective epidemiological research studies in Iran
PCA: Principal component analysis
WHR: Waist-to-hip ratio
DBP: Diastolic blood pressure
SBP: Systolic blood pressure

## References

[1] Fisher ND, Curfman G (2018). Hypertension—a public health challenge of global proportions. JAMA.

[2] Mills KT, Stefanescu A, He J (2020). The global epidemiology of hypertension. Nat Rev Nephrol.

[3] Mirzaei M, Moayedallaie S, Jabbari L, Mohammadi M (2016). Prevalence of Hypertension in Iran 1980–2012: a systematic review. J Tehran Heart Cent.

[4] Collier SR, Landram MJ (2012). Treatment of prehypertension: lifestyle and/or medication. Vasc Health Risk Manag.

[5] Lima R, Wofford M, Reckelhoff JF (2012). Hypertension in postmenopausal women. Curr Hypertens Rep.

[6] Khansari N, Shakiba Y, Mahmoudi M (2009). Chronic inflammation and oxidative stress as a major cause of age-related diseases and cancer. Recent Pat Inflamm Allergy Drug Discov.

[7] Siti HN, Kamisah Y, Kamsiah J (2015). The role of oxidative stress, antioxidants and vascular inflammation in cardiovascular disease (a review). Vascul Pharmacol.

[8] Pisoschi AM, Pop A (2015). The role of antioxidants in the chemistry of oxidative stress: a review. Eur J Med Chem.

[9] Floegel A, Kim D-O, Chung S-J, Song WO, Fernandez ML, Bruno RS (2010). Development and validation of an algorithm to establish a total antioxidant capacity database of the US diet. Int J Food Sci Nutr.

[10] Li B, Li F, Wang L, Zhang D (2016). Fruit and vegetables consumption and risk of hypertension: a meta-analysis. J Clin Hypertens.

[11] Sabião TS, Bressan J, Pimenta AM, Hermsdorff HHM, Oliveira FL, Mendonça RD (2021). Influence of dietary total antioxidant capacity on the association between smoking and hypertension in Brazilian graduates (CUME project). Nutr Metab Cardiovasc Dis.

[12] Wu L, Sun D, He Y (2016). Fruit and vegetables consumption and incident hypertension: dose–response meta-analysis of prospective cohort studies. J Hum Hypertens.

[13] Villaverde P, Lajous M, MacDonald C-J, Fagherazzi G, Bonnet F, Boutron-Ruault M-C (2019). High dietary total antioxidant capacity is associated with a reduced risk of hypertension in French women. Nutr J.

[14] Martins Gregório B, Benchimol De Souza D, de Morais A, Nascimento F, Matta L, Fernandes-Santos C (2016). The potential role of antioxidants in metabolic syndrome. Curr Pharm Des.

[15] Ayeneh Pour A, Moradinazar M, Samadi M, Hamzeh B, Najafi F, Karimi S (2020). Association of dietary inflammatory index with cardiovascular disease in Kurdish adults: results of a prospective study on Ravansar non-communicable diseases. BMC Cardiovasc Disord.

[16] Darbandi M, Hamzeh B, Ayenepour A, Rezaeian S, Najafi F, Shakiba E (2021). Anti-inflammatory diet consumption reduced fatty liver indices. Sci Rep.

[17] Jam SA, Rezaeian S, Najafi F, Hamzeh B, Shakiba E, Moradinazar M (2022). Association of a pro-inflammatory diet with type 2 diabetes and hypertension: results from the Ravansar non-communicable diseases cohort study. Arch Public Health.

[18] Pasdar Y, Najafi F, Moradinazar M, Shakiba E, Karim H, Hamzeh B (2019). Cohort profile: ravansar non-communicable disease cohort study: the first cohort study in a Kurdish population. Int J Epidemiol.

[19] Aadahl M, Jørgensen T (2003). Validation of a new self-report instrument for measuring physical activity. Med Sci Sports Exerc.

[20] Moradi S, Pasdar Y, Hamzeh B, Najafi F, Nachvak SM, Mostafai R (2018). Comparison of 3 nutritional questionnaires to determine energy intake accuracy in Iranian adults. Clin Nutr Res.

[21] Haytowitz DB, Bhagwat S (2010). USDA database for the oxygen radical absorbance capacity (ORAC) of selected foods, release 2. US Dep Agric.

[22] Wu X, Beecher GR, Holden JM, Haytowitz DB, Gebhardt SE, Prior RL (2004). Lipophilic and hydrophilic antioxidant capacities of common foods in the United States. J Agric Food Chem.

[23] Rajati F, Hamzeh B, Pasdar Y, Safari R, Moradinazar M, Shakiba E (2019). Prevalence, awareness, treatment, and control of hypertension and their determinants: results from the first cohort of non-communicable diseases in a Kurdish settlement. Sci Rep.

[24] Wang Y, Chun OK, Song WO (2013). Plasma and dietary antioxidant status as cardiovascular disease risk factors: a review of human studies. Nutrients.

[25] Ahmad KA, Yuan Yuan D, Nawaz W, Ze H, Zhuo CX, Talal B (2017). Antioxidant therapy for management of oxidative stress induced hypertension. Free Radic Res.

[26] Czernichow S, Bertrais S, Blacher J, Galan P, Briançon S, Favier A (2005). Effect of supplementation with antioxidants upon long-term risk of hypertension in the SU. VI. MAX study: association with plasma antioxidant levels. J Hypertens.

[27] Sorriento D, De Luca N, Trimarco B, Iaccarino G (2018). The antioxidant therapy: new insights in the treatment of hypertension. Front Physiol.

[28] Yamada T, Nagata K, Cheng XW, Obata K, Saka M, Miyachi M (2009). Long-term administration of nifedipine attenuates cardiac remodeling and diastolic heart failure in hypertensive rats. Eur J Pharmacol.

[29] Rizzi E, Guimaraes DA, Ceron CS, Prado CM, Pinheiro LC, Martins-Oliveira A (2014). β1-Adrenergic blockers exert antioxidant effects, reduce matrix metalloproteinase activity, and improve renovascular hypertension-induced cardiac hypertrophy. Free Radic Biol Med.

[30] Otsuka T, Takada H, Nishiyama Y, Kodani E, Saiki Y, Kato K (2016). Dyslipidemia and the risk of developing hypertension in a working-age male population. J Am Heart Assoc.

[31] Kashyap MK, Yadav V, Sherawat BS, Jain S, Kumari S, Khullar M (2005). Different antioxidants status, total antioxidant power and free radicals in essential hypertension. Mol Cell Biochem.

